# Practical Notes and Formulæ

**Published:** 1882-06-20

**Authors:** 


### PRACTICAL NOTES AND FORMULÆ.

**Cinchonic Mixtures.**—

RHEUMATIC MIXTURES.

| | |
|---|---|
| Sulphate of quinia | 30 grains, |
| Iodide of potassium | 15 " |
| Dilute sulphuric acid | 15 drops, |
| Distilled water | 4 ounces, |
| Simple syrup | 1 drachm. |

Mix. Dose, a teaspoonful every two hours.

SWEET QUININE MIXTURE FOR CHILDREN.

| | |
|---|---|
| Sulphate of quinia | 15 grains, |
| Cold infusion of coffee | 3 ounces, |
| Syrup of chloroform | 12 drachms. |

SYRUP OF CHLOROFORM.

| | |
|---|---|
| Chloroform | 20 drops, |
| Alcohol | 90 minims, |
| Simple syrup | 3 ounces. |

Mix the chloroform and the alcohol, and to the solution add the simple syrup. A good addition to bitter mixtures and drops.

FEVER SYRUP FOR POOR PEOPLE.

| | |
|---|---|
| Quinoidine | 30 grains, |
| Citric acid | 05 " |
| Alcohol | 100 " |
| Syrup of coffee | 2½ ounces. |

Dissolve the acid and the alkaloid in the alcohol, and add the syrup in the solution.—*Monthly Review of Med.*

**Treatment of Eczema.**—In chronic eczema, especially in infants, and in eczema of the face, Dr. Lassar recommends an ointment. The formula for an ointment in eczema of the face, which cannot be rubbed off during sleep, is—

| | | |
|---|---|---|
| Salicylic acid | ʒss; | 2.00 gm., |
| Oxide of zinc, Starch | aa ʒvjss; | 25.00 gm., |
| Vaseline | ʒxiij; | 50.00 gm. |

**Diarrhœa.**—In certain cases of diarrhœa characterized by a want of intestinal tonicity, A. W. Hagenbach, M. D., (Chicago Med. Jour. and Exam.) has used the following with marked success—

| | | |
|---|---|---|
| ℞ Olei terebinth | fl. ʒij; | 8.00 fl. gm., |
| Tinct. opii | fl. ʒiij; | 12.00 fl. gm., |
| Syrup kramariæ | fl. ℥ ij; | 60.00 fl. gm., |
| Aquæ puræ, ad | fl. ℥iv; | 120.00 fl. gm., |

M. Emulsif. Sig. A teaspoonful every three or four hours.

**Treatment of Uterine Fibroids.**—Fibroids of the uterus may often be successfully treated by the use of suppositories of ergotin, made according to the following formula:

| | |
|---|---|
| Ergotin | gr. 1-12; 0.005 gm., |
| Coca butter | gr. xxiij; 1.50 gm. |
| Vaseline | q. s. |

For one suppository.

These suppositories are of equal use in cases of menorrhagia, metrorrhagia, and chronic metritis.—*Le Progres Medical: Lond. Pract.*

**Hope's Camphor Mixture.**—

| | | |
|---|---|---|
| ℞ | Acidi nitrosi | 1 drachm, |
| | Tinct. opii | 40 drops, |
| | Aquæ camph | 8 ounces. |

M. Dose, one or two tablespoonfuls in diarrhœa or dysentery every two to four hours.

**Pills for Constipation.**—*Lay.*

| | | |
|---|---|---|
| ℞ | Quiniæ sulphatis | |
| | Piperinæ | aa, gr. xv, |
| | Hydrarg. submuriat | gr. xii, |
| | Ext. nucis vomic | gr. iv. |
| | Ft pills no. xxx. | |

M. S. One pill morning and evening.—*N. O. Med. Jour.*

**Treatment of Dysentery.**—*Defize.*

| | | |
|---|---|---|
| ℞ | Decocti cinchonæ | fl. ℥vi, |
| | Potass. chloratis | ℥ss. *M.* |

S. All to be taken in the course of twenty-four hours.—*Ibid.*

**Blue Ointment for Ring-Worm.**—*Claudat.*

| | | |
|---|---|---|
| ℞ | Adipis | ʒvi, |
| | Glycerinæ, | fl. ʒii, |
| | Sodii carbonat | ʒi, |
| | Calcis pulv | ʒss, |
| | Carbonis (liq.) pulv | ℥iss. |

M. S. Before applying this salve remove scabs by using starch poultices. Treatment should be kept up two or three months, in cases of tenia tonsurans.—*Ibid.*

**Treatment of Laryngeal Phthisis.**—*Cadier.*

Pencil the ulcerated surfaces with the following mixture:

| | | |
|---|---|---|
| ℞ | Glycerinæ | fl. ℥i—℥ii. |
| | Alcohol, | fl. ʒv—ʒviii, |
| | Creasoti | m. xv. |

M.—*N. O. Med. Jour.*

**Oil of Turpentine in Mixtures.**—Oil of turpetine is a medicament that few patients can take without disgust. Ordinary sulphuric ether, luckily, has the property of modifying its persistently unpleasant flavor. The following mixture has been found to be very beneficial in vesical catarrh, neuralgia, and sciatica:

Oil of turpentine .......................... 2 drachms,
Sulphuric ether .......................... 1 drachm.

Mix by shaking violently, and add

Syrup of orange flowers .................. 1 ounce,

Water ..................................... 4 ounces.

A teaspoonful to be taken every two hours.—*Drug. Cir.*

**Hardy's Ointment.**—This ointment, used in France to prevent falling off of the hair, is given as follows by Bourchardat (Ann. de Therap.):

Beef fat ................................ 17 drachms.
Castor oil .............................. 6 "
Gallic acid ............................. 30 grains,
Vanilline, .............................. q. s.

—*Druggist's Circular.*

**Pills for Dyspepsia.**—

Diastase ................................ 10 grains,
Pepsine ................................. 50 "
Extract of gentian ...................... 50 "
Tartaric acid ........................... 50 "
Powdered rheubarb ....................... 50 "
Gentian, sufficient. Divide into three-grain pills.

Dose, two to three pills during meals or shortly before.—*Drug. Circular.*

**Arsenical Treatment of Chorea.**—M. Siredey, we read in the Revue de Therap. Med et Chirurg., has long had recourse to arsenic in the treatment of rheumatismal chorea, and has had good results from this medication. He uses the solution of Bondin :—

℞ Ac. arsenious, ................................ gr. xv
Aquæ destill., ................................ Oij.

M. This is allowed to boil for about a quarter of an hour, and is then ready for use. The extreme dilution of the solution is favorable for its use among children. For a child from six to ten years of age, about one drachm and a half may be given daily, in divided doses, in sweetened water. In this way the medicament is generally well tolerated.

Dr. Sottmann, of the Breslan Clinic, constantly prescribes the following solution in chorea :

℞ Liquor Fowlere, ............................ m iv
Aquæ, ........................................ ℥ ij.

M. This quantity to be taken in divided doses daily. He generally obtains a complete cure in from 16 to 21 days.

In some cases he adds from 8 to 15 grains of chloral to the prescription. He has generally found that anæmic children, and those of hereditary nervous disposition, bear this form of medication well.—*Med. & Sur. Reporter.*

**New Treatment for Vaginitis.**—M. Terrillon proposes a method of treatment which eonsists essentially in the introduction into the vagina of the following ointment :

| | | |
|---|---|---|
| ℞ | Ac. tannic,........................... | 50 grams. |
| | Amyli,.................................. | 150 grams. |
| | Ung. petrolei,.......................... | 150 grams |

M. This ointment is placed in a sort of speculum, so arranged that the ointment can be forced out as the instrument is withdrawn from the vagina. If the vulvar opening is large a small tampon of cotton may be introduced.

Generally from fifteen to twenty grams of the unguent is sufficient at one application, and it need not be repeated for seven or eight days.—*Med. Surg. Record.*

**Boils.**—Boracic acid applied to boils before or after incision, says an exchange, will promptly arrest their development.

**Quinidia Combination for Intermittents.**—Reed recommends the following formula in the treatment of intermittents :

| | | |
|---|---|---|
| ℞ | Quinidiæ sulph............................ | grs. xxx |
| | Oleo-resinæ capsici........................ | grs. iij |
| | Morphiæ sulph.............................. | gr. j |
| | Syrupi..................................... | q. s |

M. Ft. pill., No. x. Sig. One every three hours.

He says that if a cholagogue cathartic be given at the start, and a full opiate (preferably a hypodermic injection of morphia) an hour before the expected paroxysm, this combination has been uniformly successful in his hands, even in most obstinate cases of intermittents.

In cases of malarial cachexia he finds that many recover under the influence of sea air, without any medication. When the latter is necessary he has seen excellent results from small doses of nux vomica combined with the sixteenth of a grain of podophyllin, or one-fourth of a grain of blue pill, and repeat every four or six hours.—*Med. and Surg. Rept.* Jan. 10, '80.

**Hot Water for the Heart.**—Dr. A. Paggi records the following observation: He states that in Paris he saw a case in which, under the inhalation of chloroform, the heart ceased to beat, and artificial respiration for ten minutes failed to restore circulation, when Dr. Labbe dipped a cloth in boiling water and applied it in the region of the heart, with the result of immediately restoring the action of that organ.—*N. Y. Med. Times.*

**Quinia in the Treatment of Cholera Infantum.**—Dr. Otis T. Manson, of Richmond, says he has not lost a case of this disease since employing quinine in conjunction with calomel. Quinine is given until full physiological effects are obtained.—*Cincinnati Obstet. Journal.*

